# A Tissue Section-Based Mid-Infrared Spectroscopical Analysis of Salivary Gland Tumors Based on Enzymatic Deglycosylation

**DOI:** 10.3390/cancers17091545

**Published:** 2025-05-01

**Authors:** Julie Wellens, Robin Vanroose, Sander De Bruyne, Hubert Vermeersch, Benjamin Denoiseux, David Creytens, Joris Delanghe, Marijn M. Speeckaert, Renaat Coopman

**Affiliations:** 1 Department of Biomedical Sciences, Ghent University, 9000 Ghent, Belgium; 2 Department of Oro-Maxillofacial, Plastic, Reconstructive and Aesthetic Surgery, Ghent University Hospital, 9000 Ghent, Belgium; robin.vanroose@ugent.be (R.V.); hubert.vermeersch@ugent.be (H.V.); benjamin.denoiseux@uzgent.be (B.D.); renaat.coopman@ugent.be (R.C.); 3 Department of Diagnostic Sciences, Ghent University Hospital, 9000 Ghent, Belgium; sanderr.debruyne@ugent.be (S.D.B.); joris.delanghe@ugent.be (J.D.); 4 Department of Laboratory Medicine, Jessa Hospital, 3500 Hasselt, Belgium; 5 Department of Diagnostic Sciences, Cancer Research Institute Gent (CRIG), Ghent University, 9000 Ghent, Belgium; david.creytens@uzgent.be; 6 Department of Pathology, Ghent University Hospital, 9000 Ghent, Belgium; 7 Research Foundation-Flanders (FWO), 1000 Brussels, Belgium; 8 Department of Clinical Chemistry, Microbiology and Immunology, Ghent University, 9000 Ghent, Belgium; 9 Department of Nephrology, Ghent University Hospital, 9000 Ghent, Belgium

**Keywords:** ATR-FTIR spectroscopy, enzymatic deglycosylation, glycosylation biomarkers, mucin-associated glycoproteins, salivary gland tumors

## Abstract

Background/Objectives: Salivary gland tumors (SGTs) are a rare and histologically heterogeneous group of neoplasms that are challenging to diagnose due to phenotypic heterogeneity and overlapping histomorphological markers. Accurate diagnosis is required for clinical management, particularly in unusual subtypes. The objective of this study was to ascertain whether attenuated total reflectance–Fourier transform infrared (ATR-FTIR) spectroscopy, in combination with enzymatic deglycosylation, would be useful in SGT classification by detecting glycosylation-related metabolic variations. Methods: 155 tissue sections, consisting of 80 SGTs and 75 controls, were analyzed. ATR-FTIR spectroscopy was used to record the mid-infrared (MIR) spectra (4000–400 cm^−1^) of enzymatically untreated and deglycosylated samples. Spectral data were preprocessed and analyzed by principal component analysis (PCA) and orthogonal partial least squares-discriminant analysis (OPLS-DA). Enzymatic deglycosylation focused on sialic acid and fucose residues with α2-3,6,8 neuraminidase, α1-2,4,6 fucosidase O, and α1-3,4 fucosidase. Results: Tumor and control samples were discriminated with an OPLS-DA model, achieving an accuracy of 81.9% (78.7% for controls and 85.0% for tumors), especially in the glycosylation-relevant spectral range (850–1250 cm^−1^). Classification between benign and malignant tumors was more challenging, with an accuracy of 70.0% (72.5% for benign and 67.5% for malignant cases). Enzymatic deglycosylation resulted in detectable changes in the MIR spectra, confirming the contribution of glycosylation to tumor-specific signatures. Benign vs. malignant tumor discrimination was still poor and was not much enhanced in the sense of incorporating glycosylation-specific regions. Conclusions: ATR-FTIR spectroscopy coupled with enzymatic deglycosylation can distinguish tumor and control tissues based on glycan-associated spectral differences. Application of the technique to benign/malignant SGT discrimination is hampered by spectral overlap and tumor heterogeneity. Further research will be necessary to explore other clustering algorithms and larger and more homogeneous datasets for improved diagnostic accuracy.

## Simple Summary

Salivary gland tumors are rare and can be difficult to diagnose accurately because of their wide variety and overlapping features. Traditional methods using microscope-based tissue analysis sometimes struggle to clearly distinguish between benign and malignant tumors. In this study, we explored a new approach that uses infrared light to detect chemical changes in tumor tissue, specifically looking at sugar structures on the surface of cells that tend to change when tumors become cancerous. We also took some of the tissue samples and treated them with enzymes to eliminate these sugar structures. Our results indicate that this technique can be used to distinguish between tumor tissue and normal tissue, but not as readily to distinguish between various types of tumors. This could assist pathologists in making more precise diagnoses and could also translate into quicker or more precise cancer detection.

## 1. Introduction

Salivary gland tumors (SGTs) are a heterogeneous and rare group of benign and malignant tumors. They represent 3% of all head and neck cancers [1], and 80–85% of SGTs arise in the parotid gland [2]. Mucoepidermoid carcinoma (MEC) and adenoid cystic carcinoma (ADCC) are the most common malignant subtypes, whereas pleomorphic adenoma (PA) is the most frequent benign tumor. Although SGTs have an overall malignancy rate of 20%, this percentage can vary depending on the site of occurrence. Tumors of the minor salivary glands, for example, are malignant in approximately 50% of cases, whereas SGTs of the sublingual gland are almost exclusively malignant [3].

Currently, diagnosis relies on histomorphological microscopic analyses. However, the rarity, indolent nature, and phenotypic heterogeneity of SGTs limits pathologists’ familiarity with uncommon subtypes. Consequently, this leads to significant inter-observer variability and challenges in achieving accurate histological diagnosis. This discrepancy is particularly relevant in rare malignant subtypes, where achieving high diagnostic accuracy is crucial because of the significant impact of false-negative or false-positive results [1]. Histomorphology, using H&E or immunohistological stains, is the gold standard for SGT diagnosis but requires sufficient tissue [1,4]. Small core biopsies or fine needle aspiration cytology (FNAC) may not capture intra-tumor heterogeneity, leading to inter-observer variability, especially given the numerous subtypes. Immunohistochemistry can aid in subtype identification, although immunophenotypes may overlap. Prognosis and treatment depend on the tumor’s oncological status, with malignancy defined by invasion, and metastasis characterized by rapid growth, irregular borders, and the spread via blood or the lymphatic systems [1,5,6].

A correct preoperative diagnosis is paramount. Therefore, it may be relevant to consider the introduction of adjuvant techniques to aid pathologists in the classification of malignant and benign subtypes in cases where histomorphology does not provide a clear answer. Alternative biomarkers for malignancy, such as tumor surface glycosylation, should be considered. During the malignant transformation of cells, tumors cause changes in surface glycosylation (an enzyme-mediated, non-random process) to aid in development, progression, and immune escape. Sialylation and fucosylation are especially significant in salivary gland tumorigenesis. Increased tumor growth and mechanisms of immune evasion have been attributed to elevated sialylation of SGTs, whereas aberrant fucosylation patterns have been associated with malignancy and metastatic capacity. Neuraminidase preferentially eliminates terminal sialic acids, and the fucosidases cleave core and terminal fucose residues, both of which are critical modifications that are engaged in the malignant phenotype of SGTs. Accordingly, the selection of such enzymes guarantees selective removal of important glycans involved in SGT pathogenesis, enabling more effective biochemical discrimination between tumor and non-tumor tissues [7]. Salivary glycoproteins, of which mucins are the most important and abundant (26% of salivary proteins) [8], undergo posttranslational modifications on their saccharide chains, such as sialylation and core and terminal fucosylation, during malignant transformation [7]. Mucins consist of 20–55% serine, threonine, and proline residues in their protein backbone, with O-linked oligosaccharides making up 40–80% of their mass [8]. MUC1 is the most well-characterized transmembrane mucin, encoded by a gene located on chromosome 1q21-24 [9]. MUC5B and MUC7 are two of the most important secretory mucins, which provide moisture, lubrication, and protection to the cell surface from physical and chemical injury [10].

Attenuated total reflectance–Fourier transform infrared (ATR-FTIR) spectroscopy, specifically in the mid-infrared (MIR, 4000–400 cm^−1^) region, based on chemical bond vibrations, has proven to be a valuable technique for detecting biochemical changes associated with glycosylation processes [11]. FTIR is based on the principle that when a sample is irradiated with infrared (IR) light, the IR-active bonds (with a dipole moment) will only absorb photons with an identical frequency and bend, stretch, or vibrate at a specific frequency [12]. As all the bonds within the sample absorb their characteristic frequencies/wavelengths, a sample-specific IR spectrum can be acquired in which the intensity is measured at each wavelength. Within this spectrum, specific regions are linked to specific bonds. This is a user-friendly, fast, minimal-sample preparation method with a higher sensitivity and specificity compared to classic MIR spectroscopy. ATR is a sampling technique in which the sample is brought into close contact with an ATR crystal. The low penetration depth at the sample–crystal interface allows for measurements that are sample thickness-independent and ideal for highly IR-absorbing materials and for minimal sample preparation [11].

This study aimed to evaluate whether ATR-FTIR spectroscopy could detect differences in the MIR spectra of different SGT histological classes. Namely, it was tested whether clustering algorithms, based on the variability in the MIR spectra of SGT samples, are able to appoint the samples to the correct sample group. This study also aimed to determine specific IR regions within the MIR range that are associated with malignant glycosylation through enzymatic deglycosylation using α2-3,6,8 neuraminidase, α1-2,4,6 fucosidase O, and α1-3,4 fucosidase to optimize prior SGT classification models. This study advances previous FTIR studies by actively affirming the biochemical underpinning of spectral diversity through site-specific enzymatic deglycosylation. Previous FTIR studies have passively documented glycosylation-dependent spectral diversity without immediate functional confirmation. By applying neuraminidase and fucosidases to specifically cleave key glycans implicated in tumorigenesis of the salivary gland, we provide novel evidence that defined glycan structure is directly transducing diagnostic MIR spectral profiles. To our best knowledge, this is the first systematic enzymatic correlation in ATR-FTIR spectroscopy of salivary gland tumors that adds a mechanistic validation to spectral interpretations and increases the translational potential of infrared-based diagnostics.

## 2. Materials and Methods

### 2.1. Sample Collection

The SGT samples utilized in this study were collected between 2012 and 2024 and were gathered from the biobank of Ghent University Hospital. A total of 155 tissue samples, comprising salivary gland tumor samples (*n* = 80) and controls (*n* = 75) originating from patients undergoing surgery for non-tumoral diseases, were included in this study. Most of the tissue samples originated from the parotid gland (*n* = 137), but some samples were derived from the submandibular gland (*n* = 17) and palatum (*n* = 1). Control tissues were collected from patients undergoing surgery for non-tumoral diseases of the salivary gland, such as sialolithiasis or benign hyperplasia. All control tissues were histologically confirmed to be normal by a head and neck pathologist with experience. Samples that contained evidence of widespread inflammation, fibrosis, or dysplasia were not used to guarantee that spectral variations would be associated with tumor processes and not with inflammatory or reparative changes. The demographics of the study population are presented in Table 1.

In the first phase of this study, 155 samples were analyzed non-enzymatically. Next, a deglycosylation subset of thirty-six patient-derived SGTs underwent enzymatic deglycosylation. This subset represented the following subtypes: 5 adenoid cystic carcinomas, 6 mucoepidermoid carcinomas, 5 Warthin tumors, 5 epithelial–myoepithelial carcinomas, 5 pleomorphic adenomas, 5 salivary duct carcinomas, and 5 acinic cell carcinomas. Additionally, 9 control samples from patients undergoing surgery for non-tumoral conditions were included in the deglycosylation subset.

### 2.2. Sample Preparation

The tissue blocks were generated through fixation of the tissue with 10% neutral-buffered formalin for 6–48 h, followed by routine processing using a Tissue-Tek VIP (Sakura, Torrance, CA, USA) and embedding the tissue in paraffin [13]. Subtyping and characterization of the tissue sections was carried out by an experienced head and neck pathologist (D.C.) in accordance with the criteria of the 2024 WHO classification of head and neck tumors [14]. An 8 µm-thick tissue section was cut from the paraffin blocks using a microtome and placed on a glass recipient, followed by stretching on a hotplate. Next, deparaffinization was performed by submerging the samples in xylene for three cycles of 2 min and 30 s, and three sequential alcohol baths (95%, 95%, and 70%) for 1 min and 20 s each. The samples were washed for one minute and twenty seconds. The slides were not coverslipped or stained.

### 2.3. Enzymatic Digestion and Infrared (IR) Analysis

The enzymes were selected based on the glycan structures described by Zalewska et al. [8]. In total, 3 enzymes were used in this study. First, α2-3,6,8 neuraminidase was used to catalyze the hydrolysis of α2-3-, α2-6-, and α2-8-linked sialic acid residues from glycoproteins and oligosaccharides (2000 units, ref. P0720S, New England Biolabs, Ipswich, MA, USA). Second, α1-2,4,6 fucosidase O was used to catalyze the hydrolysis of terminal α1-2-, α1-4-, and α1-6-linked fucose residues from oligosaccharides (80 units, ref. P0749S, New England Biolabs, Ipswich, MA, USA). Even though this exoglycosidase can sever multiple linkages, it cleaves α1-6 bonds the most efficiently. Finally, α1-3,4 fucosidase, also an exoglycosidase, was used to catalyze the hydrolysis of terminal, non-reducing α1-3- and α1-4-linked fucose residues from oligosaccharides and glycoproteins (200 units, ref. P0769S, New England Biolabs, Ipswich, MA, USA).

All of these enzymes were delivered and mixed with the same Glycobuffer 1, including 5 mM of CaCl_2_ and 50 mM of sodium acetate at a pH of 5.5 at 25 °C, according to its company guidelines (ref. B1727SVIAL, New England Biolabs, Ipswich, MA, USA). α1-3,4 fucosidase was also delivered with bovine serum albumin (BSA), including 20 mM of Tris-HCL (pH 8.0, at 25 °C), 100 mM of KCl, 0.1 mM of EDTA, and 50% glycerol (ref. B9001S, New England Biolabs, Ipswich, MA, USA).

#### 2.3.1. Enzymatic Deglycosylation

The protocol for enzymatic deglycosylation was based on the company guidelines described by New England Biolabs [15]. From the deglycosylation sample subset, four consecutive slides were cut from each paraffin block to represent a specific condition. Each condition was treated with 200 µL of the respective treatment solution and incubated overnight at 36 °C. Treatment solutions were made according to Table 2, and the control condition was treated with 200 µL PBS (0.1 mol/L at a pH of 7.3, containing 137 mmol/L of NaCl, 2.7 mmol/L of KCl, 8.1 mmol/L of Na_2_HPO_4_, and 1.47 of mmol/L KH_2_PO_4_). The following day, samples were rinsed with PBS and left to dry.

#### 2.3.2. ATR-FTIR Spectroscopy

Over the next six days, all enzymatically treated tissue slides (four in total: 1 slide of PBS control, 1 slide of α2-3,6,8 neuraminidase, 1 slide of α1-2,4,6 fucosidase O, 1 slide of α1-3,4 fucosidase) of the deglycosylation subgroup (*n* = 45) were scanned at three different locations to allow for tissue heterogeneity, using attenuated total reflectance–Fourier transform infrared (ATR-FTIR) spectroscopy in the mid-infrared (MIR) range (4000–400 cm^−1^).

The study population (*n* = 155) non-enzymatically treated samples were scanned at only one location. The Perkin Elmer Spectrum Two ATR-FTIR, including Spectrum 10 software (Perkin Elmer, Waltham, MA, USA), was used, which has a standard resolution of four cm^−1^. To avoid interference by tissue remnants of the previous samples, a 50 mm ZnSe crystal was cleaned between each sample analysis using an alcohol solution. Background was established by scanning in the absence of a sample. After application of the tissue sample, the pressure was adjusted to 100 g. This pressure had to be comparable for each sample, as it would influence the contact between the sample and the crystal, and therefore, the spectrum.

### 2.4. Blinding and Randomization

In an attempt to minimize bias, sample handling, enzymatic treatment, spectral acquisition, and data analysis were conducted blinded. The scientists performing ATR-FTIR measurements and enzymatic treatments were unaware of the sample class (control vs. tumor, benign vs. malignant). For machine learning modeling and data preprocessing, sample IDs were coded to blind diagnostic categories until the models were finalized. Randomization of the sample processing order was used within each experimental batch to prevent systematic biases due to sample handling, measurement drift, or operator variation.

### 2.5. Data Analysis

**Figure 1:**
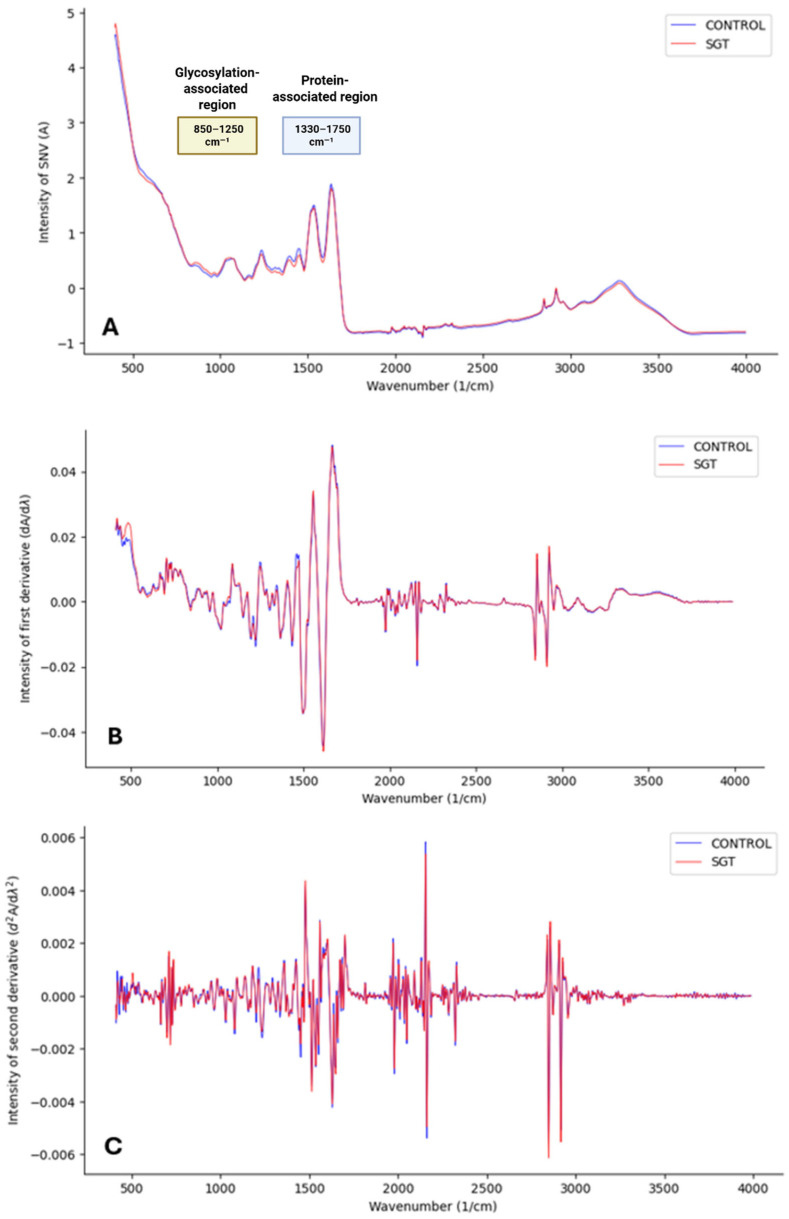
Mean MIR spectra of all controls (blue) versus all SGT samples (red) in the spectral range of 400–4000 cm^−1^. (**A**) MIR spectra after preprocessing using SNV; (**B**) MIR spectra after SNV and S-G smoothing (13 smoothing points) and 1st derivative calculation; (**C**) MIR spectra after SNV, S-G, and 2nd derivative calculation.

The spectral data, ranging from wavenumbers 400 to 4000 cm^−1^, was uploaded into the SIMCA 18 software (Sartorius Stedim Data Analytics, Goettingen, Germany) and preprocessed. To correct for scatter and baseline shifts between samples, the data was first normalized using the standard normal variate (SNV) method, where each observation is subtracted by its mean and divided by its standard deviation [16]. Subsequently, smoothing was performed using the Savitzky–Golay derivation with 13 points in the submodel. This algorithm fits a polynomial through the data points to achieve an acceptable signal-to-noise ratio while also allowing for the removal of additive and multiplicative effects through the calculation of spectral derivatives. Because taking derivatives enhances the peak resolution, the first derivative can be applied to represent the rate of change in absorbance, while the second derivative can provide insight into the rate of change in the slope of the first derivative function. However, derivative calculations, particularly the second derivative, also amplify noise (Figure 1) by introducing random variability that can influence data clustering. To mitigate these effects while maintaining spectral clarity, the preprocessing steps applied included SNV normalization, Savitzky–Golay smoothing (13 points), first-derivative calculation, and center (CTR) scaling [17]. This final scaling step ensured data comparability by correcting for systematic baseline noise.

Orthogonal partial least squares-discriminant analysis (OPLS-DA) models were used to visualize the clustering of the data. This model, just like the principal component analysis (PCA), is a tool for dimensionality reduction, meaning that the data are converted to a lower dimensional space through the calculation of new features called principal components (PCs), adding to the interpretability of complex data [18]. The horizontal component (x-axis) of the OPLS-DA score scatter plot represented variation between the groups and the vertical dimension (y-axis) will capture variation within the groups. OPLS-DA tends to overfit data, making cross-validation (CV) necessary. In this study, CV was performed by comparing the results of both the OPLS-DA and PCA models on the data [19]. We used OPLS-DA in this study because it enables the simple visualization of class differentiation by separating predictive and non-predictive (orthogonal) variance, so it is well suited to small high-dimensional data like infrared spectra. In contrast to SVM or random forests, which demand more data in order to better optimize hyperparameters without overfitting, OPLS-DA enables the straightforward explanation of class differences with in-built dimension reduction. In addition, OPLS-DA allows for direct visual inspection of the spectral features most responsible for discrimination by employing the use of coefficient plots, which is the objective of the study in biochemically explaining glycosylation-associated spectral difference.

To assess model robustness and minimize overfitting, a sevenfold cross-validation (CV) strategy was applied. The dataset was randomly partitioned into seven folds, with six folds used for model training and one fold used for validation, repeated iteratively so that each sample served once as validation. As well, permutation testing (*n* = 100) was performed by permuting labels of classes at random and retraining the model to ensure the performance of the original model in classification was considerably more than chance. This study lacked an external independent validation cohort, which is a widely recognized limitation. However, internal cross-validation and permutation testing were conducted to ensure the validity of the OPLS-DA models for this study.

## 3. Results

### 3.1. Exploration of the SGT MIR Spectra

**Figure 2:**
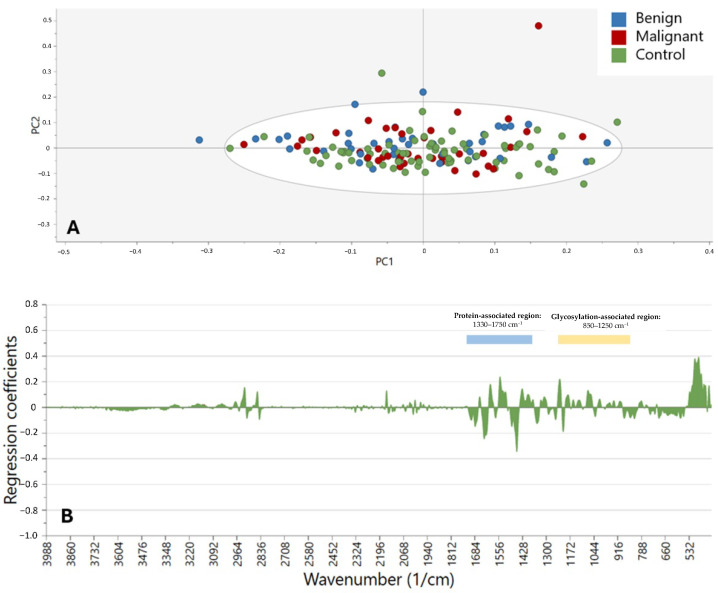
Clustering of control (green), benign (blue), and malignant (red) samples in the spectral range of 400–4000 cm^−1^. (**A**) Score plot according to the PCA. (**B**) Coefficient plot according to OPLS-DA in the spectral range of 400–4000 cm^−1^.

The MIR spectra of the 155 samples (75 controls and 80 SGTs, of which 40 were benign and 40 were malignant) were analyzed with PCA and OPLS-DA using SNV, S-G smoothing (13 smoothing points), calculation of the first derivative, and CTR scaling (Figure 1). Out of 14 samples, it was not possible to obtain a robust MIR spectrum. Figure 2 depicts the PCA and OPLS-DA models of different oncological classes (benign, malignant, and control) based on the entire MIR spectrum (400–4000 cm^−1^). The first principal component (PC) of the PCA (Figure 2A) explained 40.4% of the total variance, although the PCA model did not show a clear visual distinction between the different oncological classes. The OPLS-DA model was not able to calculate more than one PC, signifying that the variability between groups was not high enough to be used for differentiation. The wavenumbers that were most determinative of the effect of the first PC on the samples are shown in Figure 2B, showing the regression coefficient for each wavenumber. The region of 400–1750 cm^−1^ had the highest density of peaks, with some smaller peaks at 2160 cm^–1^ and in the range of 2820–2990 cm^–1^.

**Figure 3:**
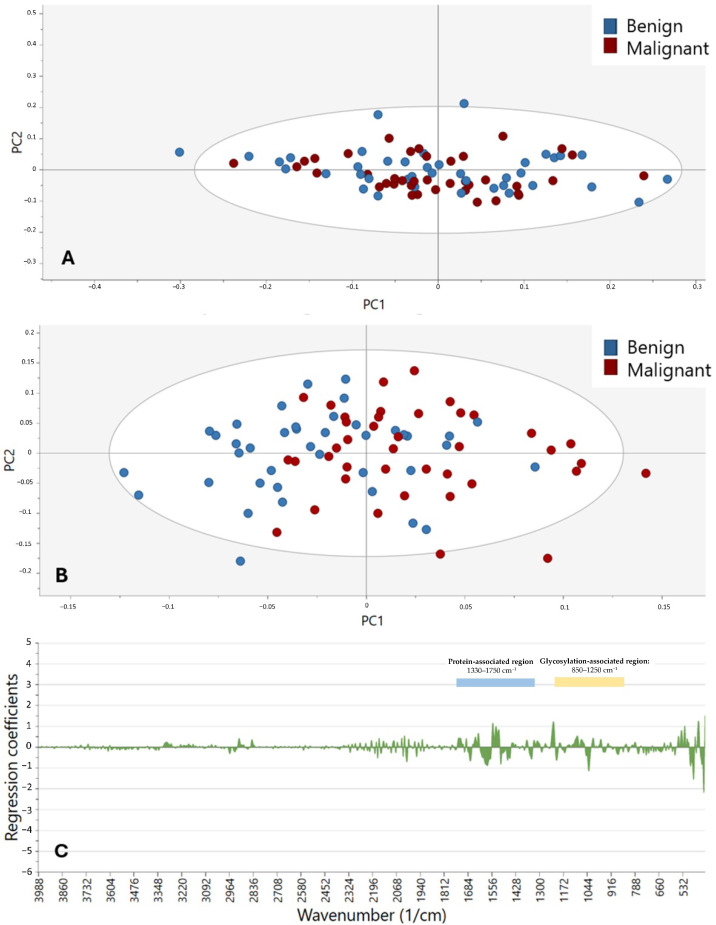
Clustering of benign (blue) and malignant (red) samples in the spectral range of 400–4000 cm^−1^. (**A**) Score plot according to PCA. (**B**) Score plot based on OPLS-DA. (**C**) Coefficient plot according to OPLS-DA showing wavenumbers of 400–4000 cm^−1^.

Considering the observed trends, OPLS-DA did not appear to be the most suitable algorithm for distinguishing between benign, malignant, and control samples. This limitation is likely due to the substantially greater variability between the control and tumor samples compared with the differences observed between benign and malignant tumors. Therefore, in Figure 3A–C, the OPLS-DA and PCA models were constructed using only the benign and malignant samples, focusing on the wavenumber range of 400–4000 cm^−1^. In this case, the OPLS-DA model (Figure 3B) could compute more than one PC, achieving a classification accuracy of 70.0% (72.5% for benign and 67.5% for malignant samples). However, as seen in Figure 3A, the PCA model did not exhibit the same degree of visual clustering between these two groups, with the first PC accounting for 39.8% of the total variability. Further analysis of the most relevant spectral regions in the coefficient plot (Figure 3C) again highlighted the key region between 400 and 1750 cm^−1^, along with smaller peaks around 1920–2320 cm^−1^ and 2825–2970 cm^−1^.

**Figure 4:**
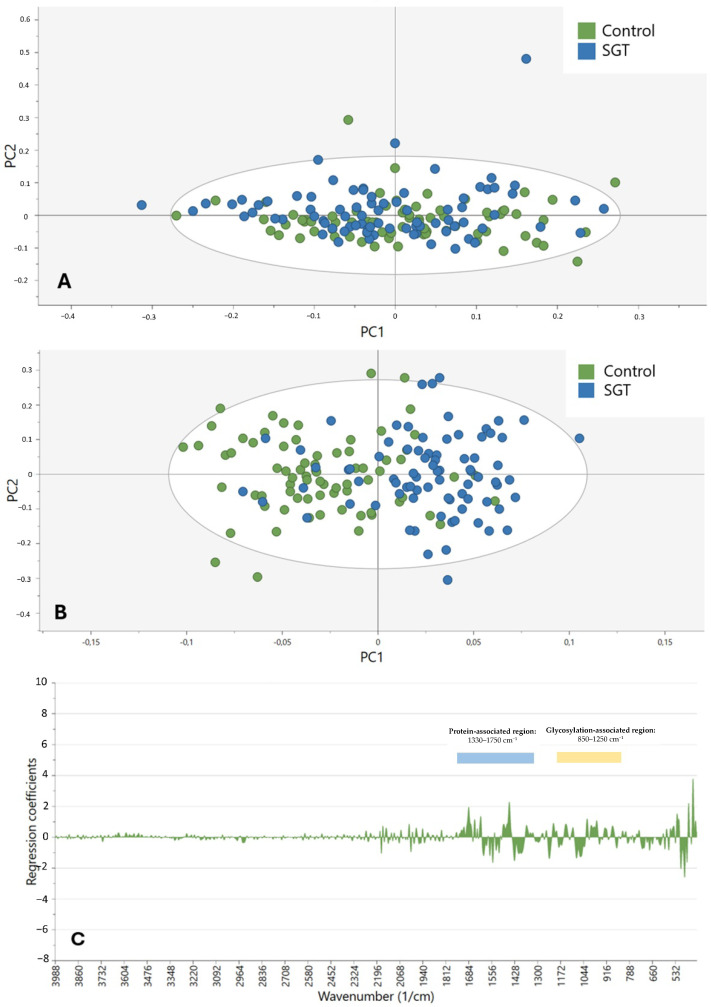
Clustering of the control (green) versus SGT (blue) samples based on a spectral range of 400–4000 cm^−1^. (**A**) Score plot based on PCA. (**B**) Score plot according to OPLS-DA. (**C**) Coefficient plot according to OPLS-DA showing wavenumbers in the range of 400–4000 cm^−1^.

The same procedure was used to compare the control and tumor (both benign and malignant) groups, as shown in Figure 4A–C. The OPLS-DA model (Figure 4B) yielded a correct classification ratio of 81.9% (78.7% for controls and 85.0% for SGTs) across all wavenumbers (400–4000 cm^−1^), with the first PC of the PCA (Figure 4A) capturing 40.0% of the total variance. Again, the visual clustering of the samples was less pronounced in the PCA model than in the OPLS-DA model. The coefficient plot (Figure 4C) highlighted the region of 400–1750 cm^−1^. Table 3 summarizes the key performance metrics (accuracy, sensitivity, and specificity) for the primary OPLS-DA classification models.

### 3.2. Enzymatic Deglycosylation of the SGT Tissue Sections and Spectral Analysis

**Figure 5:**
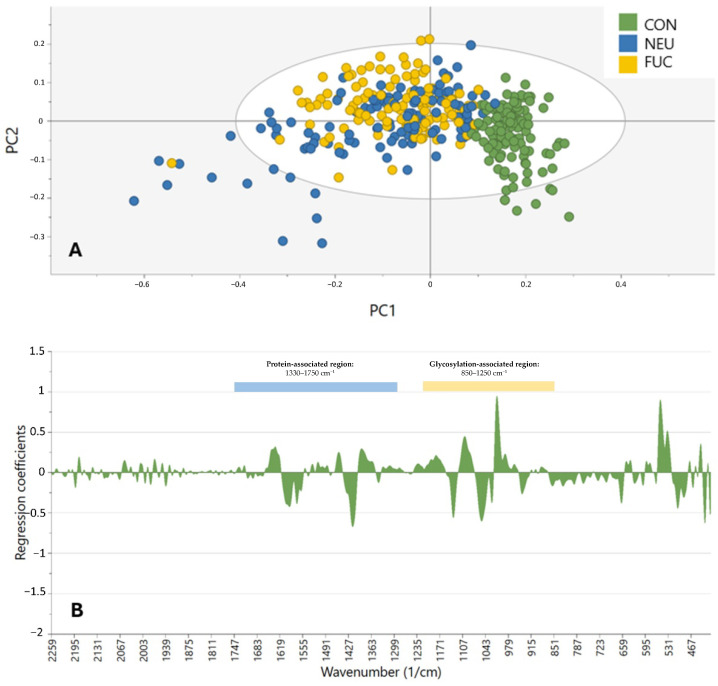
(**A**) OPLS-DA score plot of the control (CON) versus α2-3,6,8 neuraminidase (NEU) and α1-3,4 fucosidase (FUC). (**B**) Coefficient plot of the same sample groups in the range of 400–2250 cm^−1^.

**Figure 6:**
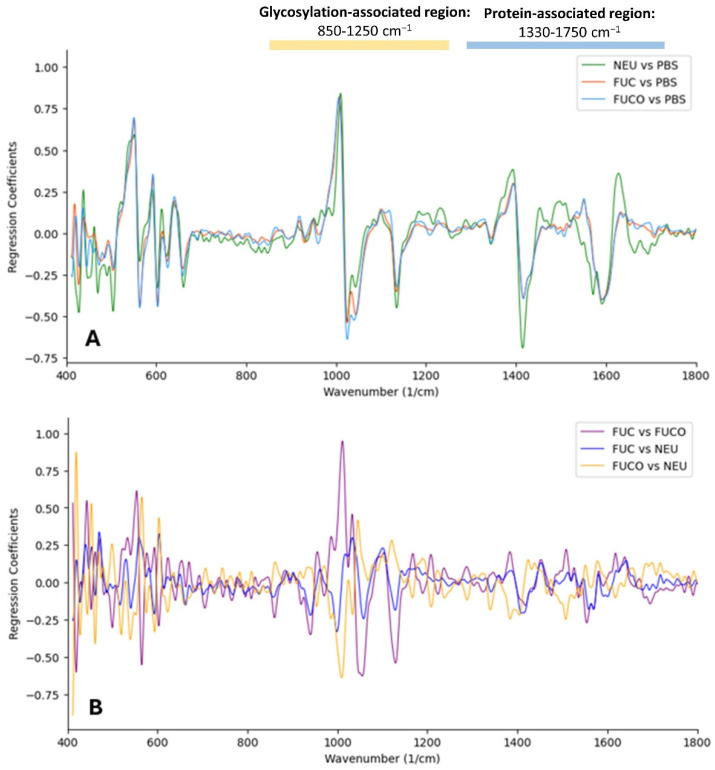
Overlay of the coefficient plots of the different treatment conditions, including PBS, α2-3,6,8 neuraminidase (NEU), α1-2,4,6 Fucosidase O (FUCO), and α1-3,4 fucosidase (FUC), in the range of 400–1800 cm^−1^. (**A**) Coefficient plots comparing the enzymatic conditions with PBS. (**B**) Coefficient plots comparing the enzymatic conditions.

Figure 5 presents the OPLS-DA model (using the same settings) for the samples (*n* = 45) treated with PBS (control), α2-3,6,8 neuraminidase, and α1-3,4 fucosidase, based on the spectral range of 400–4000 cm^−1^. To identify the key spectral regions relevant for clustering the samples that were untreated with enzymatic deglycosylation, four sets of potentially highly discriminatory spectral regions were defined, a glycosylation-associated region (850–1250 cm^−1^), a protein-associated region (1330–1750 cm^−1^), a composite region encompassing both glycosylation- and protein-associated regions, and the full mid-infrared (MIR) spectrum (1751–4000 cm^−1^) (Table 4). These regions were determined by analyzing the overlays of coefficient plots, comparing each enzymatic condition to PBS (Figure 6A) and the different enzymatic treatments to one another (Figure 6B). The specific spectral regions can be leveraged to develop refined clustering models that may improve the classification accuracy compared to models based on the full MIR spectrum.

### 3.3. Differentiating Power of Glycosylation-Associated MIR Spectral Regions

**Figure 7:**
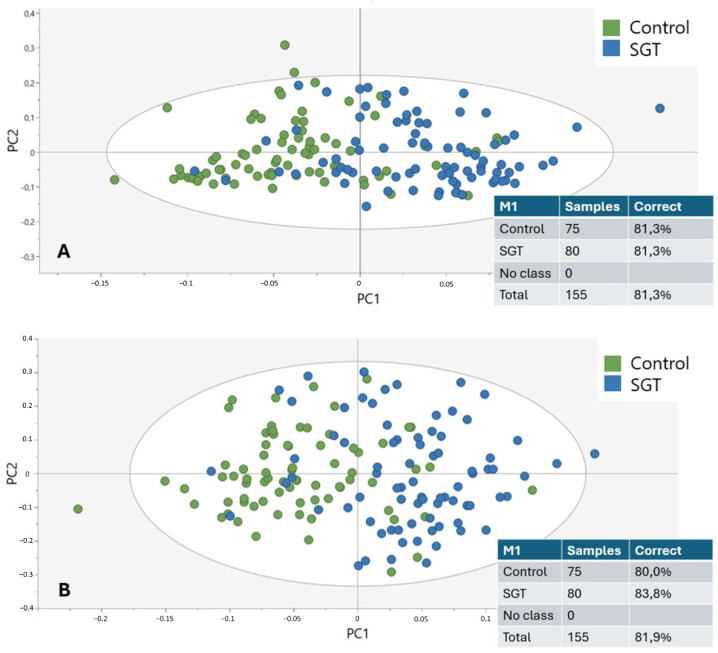
The OPLS-DA model and misclassification table of the control (green) versus salivary gland tumor (SGT, blue) samples based on specific spectral regions, defined in Table 3. (**A**) Clustering based on Set 1, including wavenumbers 850–1750 cm^−1^. (**B**) Clustering based on Set 2, including wavenumbers 850–1250 cm^−1^.

The spectral ranges detailed in Table 3 were used to create new OPLS-DA models for better differentiation among the oncological groups. Figure 7 illustrates the application of these sets to the control versus the tumor groups. OPLS-DA was able to calculate multiple PCs for set one (Figure 7A, accuracy of 81.3%) and two (Figure 7B, accuracy of 81.9%), but not for set three and four. For the malignant versus benign tumor groups, OPLS-DA could not calculate multiple PCs for any of the spectral sets.

## 4. Discussion

This study demonstrates that the diagnostic potential and feasibility of MIR spectroscopy in combination with enzymatic deglycosylation may be utilized to differentiate and characterize SGTs. By taking advantage of the biochemical specificity of ATR-FTIR spectroscopy and specifically targeting major glycosylation sites by employing sialidases and fucosidases, it was feasible to discriminate tumor from non-tumor tissue with an accuracy of up to 81.9%. This offers a critical opportunity to complement current histopathological techniques in the instance of diagnostically challenging or limited SGT types.

Among the novelties of this research is the focus given to tumor-specific glycosylation profiles, an area increasingly recognized as relevant to cancer diagnostics. Alterations in glycosylation, more specifically, sialylation and fucosylation, are involved in tumor progression, immune evasion, and cell adhesion, making them promising targets for detection and classification [7]. We confirm through our analysis that the 850–1250 cm^−1^ region, which covers the C–O and C–C stretching of carbohydrates, and 1330–1750 cm^−1^, covering the amide I and II vibrations of proteins, are enriched for discriminative features. These findings are consistent with current literature that glycans and glycoproteins have strong MIR absorbance bands in these regions [20]. While the models accurately differentiated between control and tumor tissue, the distinction between benign and malignant SGTs remained modest. This limitation would most probably be caused by both biological and technical challenges. Biologically, benign and malignant tumors often share overlapping glycan profiles, especially in low-grade malignancies or mixed histological tumors. Additionally, SGTs are highly heterogeneous, with more than 20 subtypes showing varying behaviors and biochemical compositions, hence complicating global classification even more [2]. Technically, small subgroup sizes may have hindered spectral resolution and model performance. Similar studies have also reported the same challenges. For instance, NIR spectroscopy of paraffin-embedded SGTs has shown very little difference between malignant and benign cases [13]. Compared to this, other neoplasms, such as breast and colon, have had improved stratification with FTIR since malignancy is characterized by more evident biochemical changes [21,22]. Our findings show that, in SGTs, MIR spectral alterations are more driven by tumor vs. non-tumor distinctions than by tumor grade or behavior. A further clinical application of this method can be in the diagnosis of challenging cases, such as carcinoma ex pleomorphic adenoma (Ca-ex-PA). This malignant transformation of a benign pleomorphic adenoma remains difficult to identify with routine histology alone, particularly in early or minimally invasive stages. Current research focuses on the diagnostic and management problems of giant carcinoma ex pleomorphic adenoma [23] and the need for adjunct molecular techniques. ATR-FTIR spectroscopy with enzymatic deglycosylation can potentially provide an additional level of diagnosis by detecting biochemical changes associated with malignant transformation. Translation of such molecular methodologies into the clinical environment is set to fundamentally enhance personalized treatment plans, optimizing early diagnosis, surgical planning, and prognostic stratification of intricate salivary gland neoplasms. Aside from its use in diagnosis, ATR-FTIR spectroscopy has applied advantages for clinical use. The technique is relatively fast, with individual spectral acquisition taking typically less than 5 min per sample, and requires minimal sample preparation compared to routine immunohistochemistry or molecular analysis. ATR-FTIR instrumentation costs are comparable to other lab equipment, and the method does not involve repeat antibody or reagent expenses. In addition, acquisition can be automated and is highly suited to high-throughput analysis in a pathology laboratory. However, optimization of off-the-shelf methods, operator training, and potential clinical validation studies will need to take place before clinical use becomes widespread.

An additional innovation in our study is the application of enzymatic deglycosylation prior to spectral acquisition. The treatment with neuraminidase and fucosidases removed key terminal glycans, allowing us to confirm that glycosylation is indeed a primary contributor to spectral variation in SGTs. This was evident in the improved clustering of deglycosylated samples compared to PBS-treated controls, especially in the glycan-dense 1000–1200 cm^−1^ band. Such enzymatic pretreatment is not yet exploited in FTIR literature and provides a biochemical validation procedure in keeping with spectral findings. However, care needs to be taken in interpreting protein-associated spectral modifications due to deglycosylation. The retention of strong amide I and II signals in some samples may be due to the residual presence of enzymes, as the enzymes themselves are glycoproteins. Alternatively, deglycosylation might have induced conformational alterations in glycoproteins, exposing buried functional groups and altering IR absorbance. These impacts were strongest between 1330 and 1750 cm^−1^, and have been previously reported in glycoprotein structure studies [24].

Methodologically, the use of OPLS-DA supported supervised discrimination after orthogonal variance elimination. This technique separates variability into predictive components, which distinguish predefined classes, and orthogonal components, which represent non-predictive variability. As a result, OPLS-DA not only determines whether data points differ but also explains why these differences occur. Unlike PCA, which is an unsupervised method, OPLS-DA is a supervised approach, meaning that class labels are known prior to clustering. However, its tendency towards overfitting, especially when used in small, high intergroup variance datasets, was a limitation, which was observed by way of discrepancy between OPLS-DA and PCA clustering. This has been observed previously in earlier metabolomics and spectroscopy-based cancer classification studies, and reinforces the importance of algorithm selection in spectral diagnosis [18]. Despite this, cross-validation and the use of multiple preprocessing steps (SNV, smoothing, derivatives, and CTR scaling) increased the robustness of our models.

Importantly, we confirmed that the inclusion of only the glycosylation-related spectral range did not significantly improve classification performance compared to the full-spectrum models. This suggests that, while glycan signatures are at the heart of tumor detection, multi-regional spectral information, including protein and lipid vibrations, may impart additional diagnostic information, showing that biochemical differences in SGTs are more than glycosylation alone. Yet, the glycan fingerprint region is the strongest individual predictor of tumor vs. non-tumor sample discrimination.

This study identifies several key areas for further research. More robust, larger, better-balanced sample sizes of different histological subtypes are required in order to maximize statistical power and facilitate subtype-specific classification. The inclusion of rarer SGTs will be especially critical to assess the generalizability of our findings. Second, alternative machine learning algorithms, such as support vector machines (SVMs), random forests, or deep learning-based classifiers, may offer superior performance compared to OPLS-DA by capturing nonlinear patterns in high-dimensional spectral data. Third, future investigations would likely need to incorporate multi-modal diagnostics, pairing FTIR spectral analysis with immunohistochemistry, genomic, or proteomic markers for optimal diagnostic sensitivity. Use of fresh-frozen or cytology-based samples might even allow such approaches to be directly translated into intraoperative or preoperative diagnostic techniques with the potential for supplying real-time information in the clinic. Lastly, the biochemical process of such observed spectral variations, particularly the role of mucins and specific glycan domains like sialic acid or fucose, would have to be explored in collaboration with aimed mass spectrometry or glycan profiling. It may even provide more mechanism-oriented information about the process wherein glycosylation is implicated in SGT pathogenesis and typing. While enzymatic deglycosylation provided early indication that glycan structure is responsible for diagnostic spectral features, further biochemical validation would be added value to such findings. Such follow-up research could encompass mass spectrometry-based glycomics for quantitatively measuring glycan profiles in SGT tissue before and after enzymatic treatment. Lectin-binding assays against particular sialylation and fucosylation motifs are also readily available to provide orthogonal validation of glycosylation patterns determined by ATR-FTIR. These orthogonal approaches would complement a mechanism-based understanding of spectral heterogeneity and enable further clinical translation of infrared-based cancer diagnostics.

There are a number of limitations in this study. First, the comparatively small sample size, particularly in each SGT subtype, limits the generalizability of the findings and might have hindered its ability to discriminate between benign and malignant tumors. The deglycosylation group had only forty-five samples with nine controls. Though this group had been established to maintain a near distribution across significant SGT subtypes, statistical power in enzymatic validation studies is weakened by the small sample number. In particular, having few control samples may have undermined our models’ ability to completely specify normal glycosylation profiles and malignant transformation modifications. Subsequent research would have to extend the cohort of deglycosylation to give more statistical strength and allow for more decisive findings regarding the contribution of individual glycans to diagnostic spectral variance. Second, the employment of paraffin-embedded tissue sections, although convenient, may contain artifacts over fresh or frozen tissue. Third, OPLS-DA application, even with cross-validation, risks overfitting, especially with small and heterogeneous data sets. An additional limitation of the current study is related to the enzymatic deglycosylation process. Although some exoglycosidases were utilized to selectively remove terminal sialic acid and fucose residues, quantitative confirmation of total deglycosylation efficiency was not made, and partial deglycosylation could introduce ambiguity in the spectral data. In addition, the cross-reactivity of enzymes, especially fucosidases with multi-linkage specificities, may lead to the undesired cleavage of other glycan structures and complicate spectral interpretation. Moreover, the sequence of washing and incubation steps required for enzymatic treatment can potentially introduce some additional spectral noise or sample variation, particularly in delicate paraffin sections. The quantitation of deglycosylation efficiency, the use of very specific enzymatic approaches, and the rigorous minimization of sample handling are required in future studies to prevent potential artifacts in spectral profiles. Larger multicentric datasets and additional machine learning algorithms must be used in future studies for the validation and generalizability of these findings.

## 5. Conclusions

The current study confirms that ATR-FTIR spectroscopy possesses the potential to detect differential variation in glycan structures, predominantly in protein-rich spectra (1330–1750 cm^−1^) and glycosylation-specific spectra (850–1250 cm^−1^). Enzymatic deglycosylation provides supportive evidence that glycan structures contribute to tumor-specific spectral patterns observed by ATR-FTIR spectroscopy. However, restricting analysis to glycan-rich areas of the spectrum did not improve classification, indicating that the entire spectral information would be needed to gain optimal discrimination. Tumor vs. control discrimination was, encouragingly, accurate (~82%), but discrimination between benign and malignant tumors remained constrained. Overall, ATR-FTIR spectroscopy, enhanced by biochemical specificity through enzymatic deglycosylation, is a promising adjunct method for objective tumor identification.

## Figures and Tables

**Table 1:** Demographic characteristics of the study population.

Study Population		All		Male		Female	
		n	Age (yrs)	n	Age (yrs)	n	Age (yrs)
Total		155	56 (18–87)	79	59 (28–79)	76	54 (18–87)
Control group		75	53 (18–75)	42	57 (28–75)	33	48 (18–74)
SGTs		80	59 (19–87)	37	61 (39–79)	43	58 (19–87)
	Benign	40	59 (37–86)	21	62 (39–79)	19	56 (37–86)
	Pleomorphic Adenoma	17	54 (37–78)	8	57 (39–78)	9	51 (37–70)
	Warthin Tumor	17	62 (52–78)	11	64 (52–78)	6	58 (52–64)
	Oncocytoma	6	68 (46–86)	2	70 (61–79)	4	66 (46–86)
	Malignant	40	59 (19–87)	16	59 (40–79)	24	60 (19–87)
	Acinic Cell Carcinoma	8	57 (34–87)	3	50 (40–58)	5	61 (34–87)
	Epithelial–Myoepithelial Carcinoma	8	61 (51–79)	4	63 (51–79)	4	59 (52–68)
	Adenoid Cystic Carcinoma	8	59 (36–72)	3	62 (58–67)	5	58 (36–72)
	Mucoepidermoid Carcinoma	8	56 (19–84)	2	49 (43–54)	6	59 (19–84)
	Salivary Duct Carcinoma	8	63 (54–78)	4	64 (56–78)	4	63 (54–72)

**Table 2:** Preparation of the treatment solution.

Condition	Enzyme Stock Concentration (U/mL)	Enzyme (µL)	Glycan Buffer (mL)	BSA (1/10 Dilution, mL)	PBS (mL)	Total Volume of the Treatment Solution (mL)
α2-3,6,8 Neuraminidase	50,000	40	1	-	9	10
α1-2,4,6 Fucosidase O	2000	40	1	-	9	10
α1-3,4 Fucosidase	4000	40	1	1	8	10

**Table 3:** Summary of model performance metrics (tumor vs. control and benign vs. malignant comparisons).

Comparison	Accuracy (%)	Sensitivity (%)	Specificity (%)
Tumor vs. control (full MIR spectrum)	81.9	85.0	87.7
Benign vs. malignant (full MIR spectrum)	70.0	67.5	72.5
Tumor vs. control (glycosylation-associated region 850–1250 cm^−1^)	81.9		
Tumor vs. control (glycosylation + protein-associated region 850–1750 cm^−1^)	81.3		

**Table 4:** Spectral ranges with high differentiating power, based on the coefficient plot overlays in Figure 5.

Set	Glycosylation-Associated Spectral Regions (cm^−1^)	Associated Functional Groups
1	850–1750	Glycosylation-associated and protein-associated spectral regions
2	850–1250	Glycosylation-associated region
3	1330–1750	Protein-associated region
4	1751–4000	Complete MIR range without noise; protein-associated or glycosylation-associated region

## Data Availability

The data presented in this study are available upon request from the corresponding author. The data are not publicly available due to the European General Data Protection Regulation.

## Abbreviations

SGT	Salivary gland tumor
FTIR	Fourier transform infrared
ATR	Attenuated total reflectance
MIR	Mid-infrared
H&E	Hematoxylin and Eosin
FNAC	Fine needle aspiration cytology
MEC	Mucoepidermoid carcinoma
ADCC	Adenoid cystic carcinoma
PA	Pleomorphic adenoma
FNAC	Fine needle aspiration cytology
OPLS-DA	Orthogonal partial least squares-discriminant analysis
PCA	Principal component analysis
PC	Principal component
SNV	Standard normal variate
S-G	Savitzky–Golay
BSA	Bovine serum albumin
PBS	Phosphate buffered saline
EDTA	Ethylenediaminetetraacetic acid
KCl	Potassium chloride
NaCl	Sodium chloride
Na_2_HPO_4_	Disodium phosphate
KH_2_PO_4_	Monopotassium phosphate
IR	Infrared
CTR	Center (scaling)
CV	Cross-validation
